# Pesticide, allergen, PCB, and lead measurements in childcare centers located on tribal lands in the Pacific Northwest, United States

**DOI:** 10.1038/s41370-023-00602-5

**Published:** 2023-09-11

**Authors:** Nicolle S. Tulve, Carry W. Croghan, Bethany L. Plewe, Holly Thompson Duffy, Katie Adams, Theresa McBride, Christopher Pace, Doug Wood, Christopher Fish

**Affiliations:** 1 United States Environmental Protection Agency, Office of Research and Development, Research Triangle Park, NC USA; 2 United States Environmental Protection Agency, Region 10, Seattle, WA USA; 3 Northwest Portland Area Indian Health Board, Portland, OR USA; 4 Indian Health Service, Portland, OR USA

**Keywords:** Chemical, Biological, Agents, Childcare, Tribal, Environmental, Soil, Dust

## Abstract

**Background:**

Children’s potential exposures to chemical and biological agents in tribal childcare centers are not well characterized.

**Objectives:**

(1) The environmental health of childcare centers in Portland Area Indian Country was characterized by measuring selected pesticides, polychlorinated biphenyls (PCBs), allergens, and lead (Pb) in outdoor soil and indoor dust. (2) We compared our results to other studies of childcare centers in both the United States and globally.

**Methods:**

At 31 tribal childcare centers in Washington, Oregon, and Idaho, we collected indoor dust and outdoor soil samples from at least one classroom, multipurpose room, and outdoor play area. Number of rooms sampled depended on facility size. Surface wipes were collected from the floor, play/work surface, and windowsill and analyzed for selected pesticides and PCBs. Vacuum samples were collected from the floor and analyzed for selected allergens. Lead was measured in surface wipes and outdoor soil collected at 11 centers. A questionnaire collected information on demographics, cleaning habits, and pesticide usage.

**Results:**

At least one pesticide was measured at all childcare centers. *cis*-Permethrin (surface wipes: 0.003–180 ng/cm^2^), *trans*-permethrin (surface wipes: 0.002–200 ng/cm^2^) and piperonyl butoxide (surface wipes: 0.001–120 ng/cm^2^) were measured in all centers. Lead was measured in most surface wipes (<0.25–14 ng/cm^2^) and all outdoor soil samples (8.4–50 mg/kg). Aroclors 1242 and 1254 were detected on indoor surfaces in three centers at very low loadings. Allergen residues were measured at very low concentrations in vacuum dust samples (Der p 1: <0.012–0.12 µg/g; Der f 1: <0.012–0.09 µg/g; Mus m 1: <0.002–10.055 µg/g). In general, we observed lower levels of chemical and biological agents than what has been reported previously.

**Significance:**

By understanding the environmental health of childcare centers, we can better understand the role of child-specific environments in promoting children’s health and well-being.

**Impact statement:**

To our knowledge, this is the first study to characterize the environmental health of tribal childcare centers in the Pacific Northwest. Combined with the information we have on childcare centers from around the world, this study expands our knowledge on young children’s potential exposures to chemical and biological agents in locations where they spend significant amounts of time.

## Introduction

Approximately 21 million children in the United States are placed in non-parental childcare each week. On average, children spend 24 h per week in center-based care [1]. For children residing on tribal lands, childcare centers are important places to: learn about their culture and native language; learn social skills; receive early childhood education; and, safely spend time while their caregivers work [2, 3]. Many chemical and biological agents may be found in the indoor environment, including childcare centers. These may occur from indoor sources (e.g., polychlorinated biphenyls [PCBs] in paints and sealants; lead [Pb] in paint), consumer product use (e.g., insecticides), presence of pets and pests (e.g., allergens), and track-in from outdoors (e.g., outdoor soil containing Pb, pesticides or PCBs). However, children’s exposures to chemical and biological agents found in and around childcare centers, and how exposure to these agents may affect their health and well-being, are not well characterized.

Childhood is a sequence of lifestages where physiology, anatomy, and behavior characterize identifiable periods of development in successive stages for each individual. Children’s physiological characteristics may influence their sensitivity to chemical and biological agents found in their everyday environment either by affecting their rate of contact with various media or by altering the exposure-uptake relationship. Children’s behaviors and the ways they interact with their environment may also influence their exposures to contaminants in their environment. As a result, children may be more vulnerable to chemical and biological agents than adults due to these differences in behavior and biology. By studying the myriad chemical and biological agents found in children’s everyday environments, we seek to improve both the environmental health of everyday locations where children spend time and their overall health and well-being.

Completed in 2001, the First National Environmental Health Survey of Child Care Centers is the only probability-based, nationally representative study of childcare centers in the United States [4–9]. A collaborative effort of the U.S. Department of Housing and Urban Development, U.S. Consumer Product Safety Commission, and the U.S. Environmental Protection Agency, this study reported on concentrations of Pb, pesticides, and allergens.

Other research groups have reported on selected chemical and biological agents such as pesticides and other persistent organic pollutants [8, 10–14], PCBs [15, 16], brominated flame retardants [17], perfluorinated compounds [17–19], metals [14, 17, 20–30], and allergens (including dust mite, cockroach, cat, dog, endotoxins, horse, fungi) [30–55] in both the indoor and outdoor childcare center environment in limited studies in the United States and globally. Collectively, this body of research shows that children may be exposed to numerous chemical and biological agents while in center-based care, yet we don’t understand how exposure to myriad chemical and biological agents may affect children’s health and well-being. Since children spend greater than 90% of their time in the indoor environment, it is important to understand the numerous stressors to which they may be exposed from all locations where they spend time (e.g., home, daycare, school). While there are no studies that characterize the totality of the indoor and outdoor childcare center environment, this study is the first to characterize several chemical and biological agents found in the childcare center environment irrespective of geographic location.

Our objectives were to (1) characterize the environmental health of childcare centers in Portland Area Indian Country (43 federally recognized tribes within the states of Idaho, Oregon, and Washington) by measuring selected pesticides, PCBs, allergens, and Pb in outdoor soil and indoor dust; and (2) compare our results with other reported studies in both the United States and globally.

## Materials and methods

### Center and room selection

All childcare centers (*n* = 43) located on tribal lands in the states of Idaho, Oregon, and Washington were eligible and recruited to participate. Childcare centers were contacted via phone and mail with information about the study. Sample collection and questionnaire administration occurred during a routine (e.g., regularly scheduled visits to the childcare centers by Indian Health Service staff) site visit to the childcare center. Each participating childcare center was given a unique participant code to ensure no center could be directly identified other than by the project lead at the Indian Health Service. Thirty-one centers agreed to participate.

For each participating childcare center, at least one classroom and outdoor play location (when bare soil was accessible) where children <6 years of age regularly spent time were sampled. Multipurpose rooms were sampled at a subset of participating childcare centers based on the usage of the multipurpose room in the center (e.g., childcare centers where the multipurpose room was routinely used by children <6 years of age). Sixty-two classrooms and nine multipurpose rooms were sampled at the thirty-one participating childcare centers. The number of classrooms sampled depended on the size of the center (7 childcare centers had one classroom sampled; 18 childcare centers had two classrooms sampled; 5 childcare centers had three classrooms sampled; 1 center had four classrooms sampled). Within each classroom and multipurpose room, surface wipe samples were collected from the floor, a play/work surface, and a windowsill. Soil samples (*n* = 33) from the outdoor play area were collected using an Incremental Sampling Methodology (ISM) approach [56].

### Questionnaire

A survey questionnaire was administered in person to the center director or designee. This questionnaire collected information on center demographics, child demographics, sampling history, playtime information, maintenance, cleaning habits, air quality, moisture/mold, and pests and pesticide usage. Room characteristic information collected is listed in the supplementary information (SI).

After obtaining permission, the field technician collected the environmental samples. Dust samples (surface wipes and vacuum) were collected in each classroom and multipurpose room and a soil sample was collected from the outdoor play area. Samples were collected from March to June 2019.

### Sample collection methods

Floor wipes, play/work surface wipes, and windowsill wipes were collected in each sampled room. The floor wipe sample was collected from either a location in the room closest to the main point of entry or exterior door closest to the room. The play/work surface wipe sample was collected from a desk or tabletop inside the classroom. The windowsill wipe was collected from the windowsill for a window that could be opened. Wipe samples were collected from hard surfaces in each sampled room.

The method used by the technician to collect a wipe sample for pesticide and PCB (collected using isopropanol-soaked gauze pads) or Pb (collected using Ghost Wipes®) analysis was the same. Briefly, using isopropanol-soaked gauze pads (3 inch X 3 inch, Medi-First, St. Louis, MO; https://mediqueproducts.com/medifirstplus/index.html) or Ghost Wipes® (Environmental Express, Charleston, SC; https://www.environmentalexpress.com/ee/s), the technician wiped the defined sampling area (area sampled = 929 cm^2^ unless otherwise measured) in an overlapping “S” pattern while applying pressure with the fingertips. The overlapping “S” pattern included the horizontal direction, folding the dust-collected side inward, then wiping in the vertical direction followed by folding the dust-collected side inward, then wiping around the perimeter of the 12 inch X 12 inch aluminum foil template. After sample collection, wipes for pesticide and PCB analysis were placed in a sample container which was then placed in a resealable plastic bag. Wipe samples for Pb analysis were placed directly into certified low metals Mod-Block digestion vials (CPI International, Inc., Santa Rosa, CA; https://www.cpiinternational.com/) and individually placed into resealable plastic bags. All wipe samples were then packed on ice in a cooler and shipped to the U.S. Environmental Protection Agency Region 10 Manchester Environmental Laboratory. When the Pb wipes were returned to the laboratory, digestion took place in these vials without further sample transfer.

To collect a soil sample, the technician used ISM [56]. When using this method to collect a composite soil sample, the sampled area is measured, gridded, and flagged with a minimum of 30 markers to mark sampling locations. After marking the sampling locations, the technician used a stainless steel coring tool (diameter 2.5 cm) to collect soil plugs. All soil plugs were put into one sampling container to create one composite sample for each outdoor play area. Composite soil samples were packed on ice in a cooler and shipped to the U.S. Environmental Protection Agency Region 10 Manchester Environmental Laboratory.

Vacuum dust samples were collected for analysis of selected allergens using a high-efficiency vacuum fitted with a DustChek™ cassette (EMLab P&K, Bothell, WA; https://www.eurofinsus.com/environment-testing/). Initially, a defined area of 3 feet by 6 feet was vacuumed for five minutes. At the completion of the initial sample collection period, the cassette was visually inspected to determine if it was a minimum of 1/3 full of dust. If not, additional sample locations in the same room were vacuumed. This step was repeated until visual inspection showed the cassette was a minimum of 1/3 full of dust. Each additional sampling location was measured and recorded. When finished, the cassette was placed in a plastic resealable bag and stored on ice in a cooler until transported to the U.S. Environmental Protection Agency Region 10 Manchester Environmental Laboratory for storage at −20 °C prior to shipping to the analysis laboratory.

### Analytical methods

#### Media preparation

##### Surface wipe samples for pesticides and PCBs

Sterile gauze pads were prepared for use by cleaning with accelerated solvent extraction using 1:1 acetone/dichloromethane (>99.9%, Honeywell, https://lab.honeywell.com/en). After drying to remove the solvent, two wipes were placed in a 2 oz. sample jar followed by addition of 5 mL isopropanol and capped. The prepared wipes were used for the collection of samples in the field as well as laboratory quality control (QC) samples. Pesticides and PCBs were analyzed from the same wipe sample aliquot.

##### Surface wipe samples for Pb

Media (GhostWipes® and Mod-Block® digestion vials) and reagents (nitric acid [69.0–70.0%], hydrochloric acid [36.5–38.0%], Baker Instra-Analyzed®, https://us.vwr.com/; and hydrogen peroxide [30%], KMG Cleanroom Grade, https://kmgchemicals.com/) were purchased certified low metals and used as received from the suppliers.

##### Soil samples for pesticides and PCBs

Sample jars (QEC Level 6, https://www.qecusa.com/) were purchased certified pre-cleaned from the supplier.

##### Soil samples for Pb

All equipment (e.g., trays, sieves, spatulas) used for ISM aliquots were washed with laboratory soap and hot water and rinsed with deionized water between uses. All other materials used for soil sampling were certified low metals from the suppliers.

#### Sample extraction/digestion

##### Surface wipe samples for pesticides and PCBs

Wipe samples were extracted following EPA Method 3580A [57]. After field sample collection and prior to laboratory analysis, surface wipe samples were spiked with solutions of pesticide surrogates (diazinon (diethyl-d10) and *cis*-permethrin (phenoxy-^13^C6)) and PCB surrogates (PCB congener 209 and tetrachlorometaxylene). Ten mL of hexane was added to each sample jar and mechanically shaken for 10 min. The hexane aliquot was then transferred to a 40 mL vial commonly used for analysis of volatile organic compounds. These steps were repeated three times (30 mL total volume). The hexane extract was reduced to approximately 10 mL on a Turbo-Vap, transferred to a 15 mL centrifuge tube, and concentrated to 2 mL on an N-EVAP. For pesticide analysis, a 1 mL sample aliquot was added to an autosampler vial, spiked with internal standard (acenaphthene-d10, chrysene-d12, naphthalene-d8, perylene-d12, and phenanthrene-d10), and analyzed. The remaining extract was subjected to sulfuric acid and florisil clean-ups for PCB analysis.

##### Soil samples for pesticides and PCBs

Soil samples were extracted following EPA Method 3541 [58]. A 10 g sample was extracted with 50:50 acetone/dichloromethane, solvent exchanged to hexane, and reduced to a final volume of 4 mL.

##### Wipe and soil samples for Pb

ISM processing at the laboratory included air drying, sieving through a 150 µm sieve, subsampling via Japanese slab cake, and fractional shoveling to an approximate aliquot size of 0.5 g. Wipe and soil samples were both digested following EPA Method 3050B [59].

#### Analysis information

##### Analysis of wipe and soil samples for pesticides

Pesticide analysis was performed following EPA Method 8270E using an Agilent 7000 gas chromatograph/mass spectrometer (GC/MS) triple quadrupole, 7890A GC, and 7693A autosampler [60]. Except for pyrethrum (a technical mixture), pesticide calibration curves were prepared at a concentration range of 1 to 500 ng/mL and were either linear or quadradic. Method reporting limits were established using 50–150% of known concentration as the acceptance criteria following EPA Methods 8270E and 8000D [60, 61]. Additional analyte specific information is listed in Table S1 and instrument conditions are described in the SI.

##### Analysis of wipe and soil samples for PCBs

Wipe and soil samples were analyzed for PCBs following EPA Method 8082A [62]. PCB analysis was performed using an Agilent 6890 GC with dual column/dual electron capture detectors and a 7683A autosampler. PCB calibration curves were prepared at a concentration range of 25 to 2500 ng/mL and were either linear or quadradic. Method reporting limits were established using 50–150% of known concentration as the acceptance criteria following EPA Methods 8270E and 8000D [60, 61]. Additional analyte specific information is listed in Table S1 and instrument conditions are described in the SI.

##### Analysis of wipe and soil samples for Pb

Digestates were analyzed on an Agilent 7700X inductively coupled plasma – mass spectrometer with an ISIS sample introduction system following EPA Method 6020B [63]. Both wipe and soil digestates were analyzed with a minimum 10X dilution. No collision or reaction gasses were used. Calibration was performed using a matrix-matched blank and multiple standards from 0.05 to 200 µg/L. Curve weighting was 1/x with a linear fit. Bismuth (m/z 209) was used as the internal standard and added using a mixing T. The reporting limits were 0.25 µg/wipe and 0.25 mg/kg in soil. At a minimum, QC check standards were analyzed every 10 samples throughout the analytical run.

#### Quality control (QC) samples

##### Quality control samples for pesticide and PCB wipe and soil samples

For each extraction batch of 20 samples or less, a method blank and laboratory control sample/laboratory control sample duplicate (LCS/LCSD) were included. Each method blank was spiked with solutions of pesticide surrogates (diazinon (diethyl-d10) and *cis*-permethrin (phenoxy-^13^C6)) and PCB surrogates (PCB congener 209 and tetrachlorometaxylene). The LCS/LCSD samples were spiked with both surrogate solutions, a pesticide solution containing all pesticides listed in Table S2 (except pyrethrum), and PCB Aroclors 1016 and 1260. Quality control results are presented in Tables S3–S6.

##### Quality control information for Pb wipe samples

For each batch of up to 20 wipe samples analyzed for Pb, QC samples included two preparation blanks (one preparation blank consisted of reagents used in the digestion and a second preparation blank included reagents and an unused Ghost Wipe®) and two laboratory control samples (LCS; one LCS consisted of reagents and a known Pb spike and another LCS consisted of reagents, an unused Ghost Wipe®, and a known Pb spike). QC samples were taken through the entire digestion and analysis process with the field samples. Quality control results are presented in Tables S3–S5.

##### Quality control information for Pb soil samples

For each batch of up to 20 soil samples analyzed for Pb, QC samples included one preparation blank (reagents used in the digestion) and two control samples (one consisted of reagents with a known Pb spike and one was a certified soil reference material). QC samples were taken through the entire digestion and analysis process with the soil samples collected in the field. In addition, ISM preparation created pairs of sand blanks made using laboratory-grade Ottawa sand; each pair consisted of one unprocessed sand aliquot and one sand aliquot that went through the ISM processing steps. The difference in Pb concentrations between these sand blanks was attributed to Pb contamination introduced during processing. Quality control results are presented in Tables S3–S5.

### Allergens

Vacuum dust samples were frozen at −20 °C and shipped to the contract laboratory for analysis. Vacuum dust samples were analyzed for Dermatophagoides pteronyssinus allergen 1 (Der p 1; dust mite), Dermatophagoides farinae allergen 1 (Der f 1; dust mite), and mouse urinary protein (Mus m 1) by the Multiplex Array for Indoor Allergens (MARIA) method and for Blattella germanica allergen 1 (Bla g 1; cockroach) by the Enzyme-Linked Immunosorbent Assay (ELISA) method. Quality control samples included field blanks (*n* = 1), laboratory blanks (*n* = 4 for Der p 1, Der f 1, and Mus m 1), and duplicates (*n* = 4 for Der p 1, Der f 1, and Mus m 1; *n* = 7 for Bla g 1). Quality control samples showed the robustness of the methods. Details of the allergen quality control samples can be found in Tables S7 and S8.

### Statistical analysis

All statistical analyses were conducted in Microsoft Excel and SAS (SAS/STAT software, version 9.4, SAS system for Windows, Cary, NC). The SAS code was reviewed by an independent expert well versed in the SAS language to ensure there were no errors in the code. All data were quality assurance reviewed to ensure data quality criteria were met.

## Results

### Childcare center demographics and questionnaire responses

Of the 43 childcare centers invited, 31 participated in this study. Table 1 summarizes the facility demographics and cleaning practices responses. These childcare centers primarily serve American Indian children under the age of 6 years (range: 12–300 children served at the time of field sampling). Nineteen childcare centers were classified as Head Start facilities. When asked about funding sources, the center director (or designee) stated multiple funding sources, including ‘the Tribe’, ‘state’, ‘federal’, and ‘private funding’. In general, the Tribes own the building and land where the childcare center exists, and also license the childcare center. Twelve childcare centers were not licensed by any entity. Most of the childcare center buildings were constructed after 1985 and only seven directors were aware of any renovations made to their buildings since 1986.

**Table 1:** Summary of facility demographics and cleaning practices from the questionnaire administered to the center director (or designee) (Total number of participating childcare centers = 31).

Characteristics	^a^Response: *N* (%)	Characteristics	^a^Response: *N* (%)
Facility demographic			
• Head Start Facility	Yes: 19 (61)	• Year Built	1986 to 2016: 23 (79)
			1978 to 1985: 0 (0)
			1960 to 1977: 3 (10)
	No: 12 (39)		
			1946 to 1959: 0 (0)
			1940 to 1945: 1 (3)
			Unknown: 4 (13)
• Facility Ownership	Tribe: 25 (81)	• Age of Oldest, Fixed Outdoor Playground Equipment	1986 to 2016: 28 (90)
	State: 1 (3)		
	Federal: 2 (6)		1978 to 1985: 1 (3)
			Unknown: 2 (6)
	Private: 3 (10)		
• Land Ownership	Tribe: 25 (81)	• Main Heating Source	Electric-Heated Forced Air (Vents): 22 (71)
			Gas-Heated Forced Air (Vents): 6 (19)
	State: 1 (3)		
			Gas Stove/Fireplace/Wall Furnace: 1 (3)
	Federal: 1 (3)		
	Private: 3 (10)		
	Unknown: 1 (3)		
			Unknown: 2 (6)
• Licensing Organization	Tribe: 13 (42)	• Drinking Water Source	Community Water System: 30 (97)
	State: 2 (6)		
	Federal: 2 (6)		
	Private: 0 (0)		
	Not Licensed: 12 (39)		
			Non-Transient, Non-Community Water System: 1 (3)
	Unknown: 2 (6)		
Cleaning practices			
• Cleaning of elevated surfaces and hard floors	Daily: 31 (100)	• Vacuuming	Daily: 29 (94)
			2–3 Times/Week: 1 (3)
			Weekly: 1 (3)
• Toys Washed	Daily: 8 (26)		
	2–3 Times/Week: 1 (3)		
	Weekly: 19 (61)		
	Monthly: 2 (6)		
	Unknown: 1 (3)		

^a^Response: N = number of individual childcare centers; % = percent of total.

The centers reported both indoor and outdoor free play time for the children. In general, tribal maintenance staff performed needed maintenance at the centers. However, it was reported that center staff, parents, and contractors also performed maintenance. Twenty-three centers reported using an HVAC system. Almost all centers reported daily cleaning of elevated surfaces, hard floors, and carpeted floors. Most centers (*n* = 19) reported weekly washing of children’s toys. Sixteen centers reported using a pesticide inside the center in the prior 12 months, whereas 14 centers reported using a pesticide outside the center in the prior 12 months.

### Chemical analytes

#### Pesticides

Table S2 lists the suite of pesticides measured in the dust and soil samples according to their pesticide class. We analyzed for 46 pesticides (13 pyrethroids; 20 organophosphates; 13 other products) with 22 pesticides detected in either a surface wipe or soil sample. In summary, *cis*- and *trans*-permethrin and piperonyl butoxide were measured in all centers. Bifenthrin was measured in both wipes and soil.

Table 2 shows the summary statistics for the pesticides with >5% detection in the wipe samples. We also included diazinon for direct comparison to Tulve et al. [8]. Piperonyl butoxide was detected in 90% of the floor wipe samples. Additionally, cypermethrin, *cis*- and *trans*-permethrin, and bifenthrin were detected in greater than 50% of the floor wipe samples. Piperonyl butoxide was detected in 82% of the play/work surface wipe samples and 81% of the windowsill wipe samples. *Cis*- and *trans*-permethrin were also detected in >50% of the play/work surface wipe samples and windowsill wipe samples. In general, the highest pesticide concentrations were measured in the floor wipe samples (e.g., *cis*- and *trans*-permethrin) and the lowest concentrations were measured in the play/work surface wipe samples.

**Table 2:** Summary statistics for selected pesticides and lead measured in floor wipe samples, play/work surface wipe samples, and windowsill wipe samples collected from the participating childcare centers.

Analyte	Number of Samples Above Reporting Limit	Percent Detect	Mean	STD	Geometric Mean	50th P	75th P	90th P	95th P	Max
**Floor Wipes**										
Pesticides (*n* = 71; ng/cm^2^)										
Piperonyl Butoxide	64	90	0.1	0.54	0.016	0.02	0.03	0.09	0.16	4.6
* trans*-Permethrin	52	73	3.8	24	0.036	0.02	0.07	0.94	2.0	200
Bifenthrin	48	68	0.3	0.84	0.022	0.02	0.10	0.94	2.0	5.8
* cis*-Permethrin	38	54	3.2	20	0.036	0.01	0.05	0.76	1.2	180
Cypermethrin	37	52	0.03	0.04	0.014	0.01	0.03	0.09	0.12	0.22
Fipronil	21	30	b	b	b	a	0.003	0.05	0.7	6.2
* lambda*-Cyhalothrin	11	15	b	b	b	a	a	0.01	0.02	7.8
Cyfluthrin	9	13	b	b	b	a	a	0.12	0.64	8.2
Deltamethrin	9	13	b	b	b	a	a	0.03	0.52	0.9
Propoxur	6	8	b	b	b	a	a	a	0.003	0.004
Chlorpyrifos	5	7	b	b	b	a	a	a	0.003	0.03
Diazinon	1	1	b	b	b	a	a	a	a	0.005
Metal (*n* = 16; ng/cm^2^)										
Lead	13	81	0.62	0.32	0.56	0.52	0.74	1.0	1.4	1.4
**Play/work surface wipes**										
Pesticides (*n* = 72; ng/cm^2^)										
Piperonyl Butoxide	59	82	0.26	1.6	0.01	0.008	0.02	0.10	0.46	12
* trans*-Permethrin	55	76	0.076	0.14	0.028	0.02	0.07	0.22	0.42	0.72
* cis*-Permethrin	42	58	0.058	0.098	0.026	0.01	0.04	0.16	0.3	0.52
Bifenthrin	30	42	b	b	b	a	0.008	0.03	0.04	0.12
Cypermethrin	27	38	b	b	b	a	0.01	0.02	0.05	0.16
Fipronil	14	19	b	b	b	a	a	0.006	0.02	0.08
Deltamethrin	7	10	b	b	b	a	a	a	0.03	0.12
Cyfluthrin	6	8	b	b	b	a	a	a	0.03	0.16
Chlorpyrifos	4	6	b	b	b	a	a	a	0.002	0.02
* lambda*-Cyhalothrin	4	6	b	b	b	a	a	a	0.01	0.1
Propoxur	4	6	b	b	b	a	a	a	0.002	0.004
Diazinon	1	1	b	b	b	a	a	a	a	0.003
Metal (*n* = 16; ng/cm^2^)										
Lead	7	44	b	b	b	a	0.58	1.2	14	14
**Windowsill wipes**										
Pesticides (*n* = 67; ng/cm^2^)										
* trans*-Permethrin	58	87	0.16	0.36	0.05	0.04	0.1	0.46	0.92	1.8
Piperonyl Butoxide	54	81	2	16	0.01	0.01	0.02	0.04	0.42	120
* cis*-Permethrin	47	70	0.12	0.26	0.04	0.02	0.06	0.34	0.72	1.2
Bifenthrin	40	60	0.16	0.9	0.01	0.01	0.04	0.24	0.4	7.4
Cypermethrin	33	49	b	b	b	a	0.01	0.04	0.3	24
Fipronil	15	22	b	b	b	a	a	0.01	0.02	16
Deltamethrin	9	13	b	b	b	a	a	0.08	3.0	10
Chlorpyrifos	6	9	b	b	b	a	a	a	0.01	0.04
Cyfluthrin	6	9	b	b	b	a	a	a	0.42	3.4
Propoxur	6	9	b	b	b	a	a	a	0.004	0.01
* lambda*-Cyhalothrin	3	4	b	b	b	a	a	a	a	0.12
Diazinon	2	3	b	b	b	a	a	a	a	0.01
Metal (*n* = 14; ng/cm^2^)										
Lead	13	93	1.8	1.8	1.2	1.2	2.2	3.6	6.8	6.8

^a^At this percentile, all values were below the reporting limit.

^b^Statistic not calculated.

Table 3 shows the summary statistics for the pesticides with >5% detection in the soil samples. We included chlorpyrifos, cyfluthrin, *cis*- and *trans*-permethrin, and diazinon for direct comparison to Tulve et al. [8]. In general, very few pesticides were measured in the soil samples and no pesticide was measured in more than 50% of the soil samples.

**Table 3:** Summary statistics for selected pesticides and lead measured in soil samples collected from the participating childcare centers.

Analyte	Number of Samples Above Reporting Limit	Percent Detect	Mean	STD	Geometric Mean	50th P	75th P	90th P	95th P	Max
Pesticides (*n* = 33; µg/kg)										
Bifenthrin	16	48	b	b	b	a	3	20	58	120
Trifluralin	8	24	b	b	b	a	a	2.4	12	30
Deltamethrin	4	12	b	b	b	a	a	2.4	24	32
Chlorpyrifos	1	3	b	b	b	a	a	a	a	42
Cyfluthrin	1	3	b	b	b	a	a	a	a	74
* cis*-Permethrin	1	3	b	b	b	a	a	a	a	2.4
* trans*-Permethrin	1	3	b	b	b	a	a	a	a	2.2
Diazinon	0	0	b	b	b	a	a	a	a	a
Metal (*n* = 12; mg/kg)										
Lead	12	100	22	12	18	16	30	34	50	50

^a^At this percentile, all values were below the reporting limit.

^b^Statistic not calculated.

#### Polychlorinated biphenyls (PCBs)

Surface wipe and soil samples were also analyzed for seven PCBs: Aroclors 1016, 1221, 1232, 1242, 1248, 1254, and 1260. Of the 210 surface wipe samples analyzed for pesticides and PCBs, three childcare centers had measurable Aroclor residues in wipe samples (floor wipes: Aroclor 1242: 0.16–0.25 ng/cm^2^; Aroclor 1254: 0.68 ng/cm^2^; play/work surface wipes: Aroclor 1242: 0.07 ng/cm^2^; Aroclor 1254: 0.12 ng/cm^2^; windowsill wipes: Aroclor 1242: 0.53 ng/cm^2^; Aroclor 1254: 0.16 ng/cm^2^). Of the 33 soil samples analyzed for pesticides and PCBs, none had detectable Aroclor residues.

#### Lead (Pb)

Summary statistics for the Pb surface wipe and soil samples are shown in Tables 2 and 3, respectively. Pb was measured above detection in 93% of the windowsill wipe samples, 81% of the floor wipe samples, and 44% of the play/work surface wipe samples. The highest Pb concentrations were measured in the play/work surface wipe samples whereas the lowest concentrations were measured in the floor wipe samples (Table 2). All soil samples (100% detect) had measurable concentrations of Pb with concentrations ranging from 8–50 mg/kg (Table 3).

#### Allergens

Summary statistics for Der p 1, Der f 1, and Mus m 1 are shown in Table 4. Mus m 1 was measured above the limit of detection in 51% of the vacuum dust samples, while the two dust mite allergens were measured above the limit of detection in 11% (Der p 1) and 3% (Der f 1) of the vacuum dust samples, respectively. Der p 1 concentrations ranged from <0.012–0.12 µg/g and Der f 1 concentrations ranged from <0.012–0.09 µg/g. Mus m 1 concentrations ranged from 0.001–10 µg/g (Table 4).

**Table 4:** Summary statistics for selected allergens measured in vacuum dust samples collected from the participating childcare centers.

Analyte^a^	Number of Samples Above MDL	Percent Detect	Mean	STD	Geometric Mean	Min	50th P	75th P	90th P	95th P	Max
Allergens (*n* = 75; µg/g)^b^											
Mus m 1	38	51	0.4	1.6	0.01	0.001	0.002	0.04	0.32	1.4	10
			0.03^c^	0.14^c^	0.002^c^	0.0002^c^	0.001^c^	0.003^c^	0.03^c^	0.1^c^	0.8^c^
Der p 1	8	11	d	d	d	e	e	e	0.01	0.06	0.12
			d	d	d	e	e	e	0.01^c^	0.03^c^	0.06^c^
Der f 1	2	3	d	d	d	e	e	e	e	e	0.09
			d	d	d	e	e	e	e	e	0.01^c^

^a^Mus m 1: mouse urinary protein; Der p 1 and Der f 1: dust mites.

^b^Measured concentrations of Bla g 1 (cockroach) were below the method detection limit in all samples.

^c^Summary statistic calculated using the 2001 conversion factors (Mus m 1: 0.91; Der p 1: 0.59; Der f 1: 0.08).

^d^Statistic not calculated.

^e^At this percentile, all values were below the method detection limit.

## Discussion

We report frequencies and surface loadings or concentrations for selected pesticides, PCBs, allergens, and Pb measured in indoor dust and outdoor soil samples collected at tribal childcare centers located in the Pacific Northwest in the United States. To the best of our knowledge, this is the first study to measure several chemical and biological agents to assess the environmental health of childcare centers serving children under 6 years of age located on tribal lands. The methods and approaches used for sample collection were adapted from the First National Environmental Health Survey of Child Care Centers so analytes collected in both studies could be compared. Additionally, we systematically surveyed the literature to compare our analyte concentrations with concentrations measured in childcare centers from around the world.

There are very few studies in the published literature that report pesticide measurements from childcare centers. We found three studies that reported chlorpyrifos loadings from surface wipe samples collected in childcare centers. One study in North Carolina reported one hard floor surface wipe had a chlorpyrifos loading of 134 ng/cm^2^ [11], while a study in South Korea reported chlorpyrifos loadings measured on surfaces (including floor mats, desks, chairs, and toys) ranging from <1–26 ng/cm^2^ [64]. Table 5 compares the chlorpyrifos loadings measured on floor wipes and play/work surface wipes between this study and the U.S. nationally representative data [8]. For this study, the maximum loading of chlorpyrifos measured from the floor wipe samples was 0.03 ng/cm^2^, compared to a maximum of 28 ng/cm^2^ from the U.S. nationally representative data. Similar differences in chlorpyrifos loadings were observed for the play/work surface wipes (maximum concentrations: 0.02 ng/cm^2^ for this study; 4.3 ng/cm^2^ from the U.S. nationally representative survey). At the time of field sample collection for the nationally representative survey, chlorpyrifos was still available for use in the U.S. marketplace. Registered uses for chlorpyrifos were significantly restricted after 2001, and all chlorpyrifos tolerances expired in February 2022 (https://www.epa.gov/ingredients-used-pesticide-products/chlorpyrifos). These data show strong evidence for the reduction in chlorpyrifos residues with elimination of uses over time.

**Table 5:** Comparison of selected pesticides measured in surface wipe samples collected at childcare centers.

Reference	Geographic Location	Number of Samples		Chemical Analytes (ng/cm^2^)					
			Sample Type	Chlorpyrifos	*cis*-Permethrin	*trans*-Permethrin	Bifenthrin	Cypermethrin	Piperonyl Butoxide
This Study^a,c^	Pacific Northwest, USA	71	Floor Wipes	0.003 (0.005); 0.03	3.2 (20); 180	3.8 (24); 200	0.3 (0.84); 5.8	0.03 (0.04); 0.22	0.1 (0.54); 4.6
		72	Play/Work Surface Wipes	0.002 (0.002); 0.02	0.06 (0.1); 0.52	0.08 (0.14); 0.72	0.01 (0.02); 0.12	0.01 (0.02); 0.16	0.26 (1.6); 12
		67	Windowsill Wipes	0.003 (0.005); 0.036	0.12 (0.26); 1.2	0.16 (0.36); 1.8	0.16 (0.9); 7.4	0.38 (2.8); 24	2 (16); 120
Tulve et al. [8]^b,d^	USA	168	Floor Wipes	0.42 (0.21); 28	0.16 (0.04); 2.8 (*n* = 167)	0.3 (0.09); 7 (*n* = 167)	0.01 (0.002); 0.27	0.26 (0.09); 22	0.30 (0.16); 11
		80	Play/Work Surface Wipes	0.19 (0.07); 4.3	1.9 (1.5); 90	4 (3.5); 220	NA	0.19 (0.12); 23	0.13 (0.09); 3.7

^a^Mean (standard deviation); maximum.

^b^Mean (standard error); maximum.

^c^*N* = 31 childcare centers.

^d^*N* = 168 childcare centers.

Table 5 also shows comparison data for several pyrethroids (*cis*- and *trans*-permethrin, bifenthrin, and cypermethrin) and piperonyl butoxide measured in surface wipe samples. When comparing mean loadings (Tables 2 and 5), our data show that *cis*- and *trans*-permethrin and bifenthrin were higher in this study than the U.S. nationally representative data. With the exception of cypermethrin, the maximum loadings reported from this study were also higher than what was observed in the nationally representative study. Many factors affect the amount and type of pesticide residue present in the indoor environment including cleaning practices, pest pressures, temperature, humidity, product availability, application types, and frequency of use. Most likely one or more of these factors explain these observations in the data.

Comparisons of pesticide measurements from soil samples collected at childcare centers are presented in Table 6. The concentrations of *cis*- and *trans*-permethrin and chlorpyrifos reported for soil samples collected in North Carolina and Ohio were below the method detection limits [10–13], whereas the mean concentrations reported in the U.S. nationally representative data [8] ranged from 5–24 µg/kg for the pesticides of interest. Of the 33 soil samples collected in this study, only one soil sample had measurable concentrations of *cis*- and *trans*-permethrin and chlorpyrifos. All pesticide concentrations measured in both the surface wipe and soil samples were below the U.S. EPA’s Regional Screening Levels. More information about the Regional Screening Levels can be found in the SI. Because all measured concentrations were below these key screening benchmarks, no immediate remedial action was required at any participating childcare center.

**Table 6:** Comparison of selected pesticides measured in soil samples collected at childcare centers in the United States.

Reference	Geographic Location	Chemical Analytes			
		Number of Samples	Chlorpyrifos (µg/kg)	*cis*-Permethrin (µg/kg)	*trans*-Permethrin (µg/kg)
This Study	Pacific Northwest, USA	33	42^a^	2.3^a^	2.1^a^
Morgan et al. [12]	North Carolina	13	NR^b^	<1.0–2.55^c^	<1.0–2.2^c^
Morgan et al. [10]	Ohio	16; 14	NR^b^	<1.0^d^	<1.0^d^
Tulve et al. [8]	USA	117	24 (14); 1200^e^	5 (1); 130^e^	6 (1); 140^e^
Morgan et al. [11]	North Carolina	13	<1.0^d^	NR^b^	NR^b^
Wilson et al. [13]	North Carolina	4	<2^d^	NA^f^	NA^f^

^a^Measurement > RL only found in one sample.

^b^NR = not reported.

^c^Range.

^d^Method detection limit.

^e^Mean (standard error); maximum.

^f^NA = not analyzed.

We found 10 studies reporting dust Pb from floor wipe samples collected in sampled childcare centers around the world (5 from the U.S. and 5 internationally). Table 7 compares our data to the data from these 10 publicly available studies. In general, our floor dust Pb loadings are less than all other studies and also below the Pb dust clearance levels found in the Toxic Substances Control Act (10 µg/ft^2^ Pb in dust from floor wipes; 100 µg/ft^2^ Pb in dust from windowsill wipes; https://www.epa.gov/lead/hazard-standards-and-clearance-levels-lead-paint-dust-and-soil-tsca-sections-402-and-403#:~:text=EPA’s%20new%20clearance%20levels%20are,the%20dust%2Dlead%20clearance%20levels). Comparison of this data with the U.S. nationally representative data shows the maximum floor dust Pb loading measured in our study is an order of magnitude lower than what was reported for the U.S. (1.4 vs. 29.6 ng/cm^2^). For those international studies where data are reported in comparable units, our reported data are orders of magnitude less than the reported concentrations. Table 7 also shows the publicly available windowsill dust Pb loadings (our study; 3 other studies from the U.S.; data from South Africa). The windowsill dust Pb loadings reported from our study are much lower than the other reported loadings.

**Table 7:** Comparison of dust lead from floors and windowsills from sampled childcare centers around the world.

Reference	Geographic Location	Floor Pb	Windowsill Pb	Units
This Study	Pacific Northwest, USA	0.62 (0.32); <0.27–1.4^a^	1.8 (1.8); <0.27–6.8^a^	ng/cm^2^
Marshall et al. [23]	Clark County, Nevada	16.5 (71.9); <21.5–1292^a^	45.73 (103.7); <43–797^a^	ng/cm^2^
HUD Report 2003 [5]	USA	1.4 (2.2); 0.65; 29.6^b^	22 (91); 2.6; 1,243^b^	ng/cm^2^
Durkee et al. [21]	Yakima Valley, Washington	0.43–6.8^c^	NS^d^	ng/cm^2^
Weismann et al. [20]	Iowa City, Iowa	<2.7–54^c^	15–18,000^c^	ng/cm^2^
Canha et al. [30]	Clermont-Ferrand and its surrounding area in the Auvergne region, France	16 (19); 9^e^	NS^d^	ng/cm^2^
John et al. [25]	Soshanguve and Pretoria East, South Africa	17.3 ^f,g^; 6.4 ^f,h^	55.8 ^f,g^; 10.0 ^f,h^	ng/cm^2^
Orlova et al. [24]	Moscow, Russia	<1700^i^	NS^d^	ng/cm^2^
Washington State DOH [29]	Six Counties, Washington	0–1000^c^	NS^d^	µg/g
Fernandez et al. [26]	Caracas Valley, Venezuela	732–1707^c^	NS^d^	µg/g
Berglund et al. [27]	Sweden	44–240^c^	NS^d^	µg/g

^a^Mean (standard deviation); range.

^b^Mean (standard deviation); median; maximum.

^c^Range.

^d^NS = not sampled.

^e^Mean (standard deviation); median.

^f^Mean.

^g^Soshanguve.

^h^Pretoria East.

^i^Detection limit.

Table 8 compares soil Pb concentrations for 12 studies (our study and 11 others) both in the U.S. and internationally. We also include the U.S. soil Pb background concentrations for the states of Washington, Oregon, and Idaho for comparison purposes. In comparing our soil Pb concentrations with soil Pb concentrations from other U.S. studies, the range of soil Pb concentrations from our study is much less than other reported concentrations. Additionally, our reported concentrations fall within the range of concentrations reported for the U.S. soil Pb background concentrations suggesting these concentrations are within the natural concentrations found in soil in the Pacific Northwest and not due to introduced Pb contamination from current or historical uses. Only Aclan et al. [28] (Manila), Haugland et al. [14] (Bergen), and John et al. [25] (Pretoria East) reported soil Pb concentrations less than those measured in this study.

**Table 8:** Comparison of soil lead concentrations from sampled childcare centers around the world and U.S. soil lead background concentrations.

Reference	Geographic Location	Soil Pb Concentration (mg/kg)
This Study	Pacific Northwest, USA	22 (12); 8.4–50^a^
Marshall et al. [23]	Clark County, Nevada	35.89 (40.77); <7–160^a^
HUD Report 2003 [5]	USA	81 (329); 28; 3582^b^
Button [22]	greater Cincinnati area (5 county region surrounding the University of Cincinnati campus), Ohio	11–990^c,d^; 17–4636^c,e^
Weismann et al. [20]	Iowa City, Iowa	<10–1100^c^
Washington State DOH [29]	Six Counties, Washington	0–1400^c^
Aclan et al. [28]	Manila, Philippines	1.12–2.59^c^
Fernandez et al. [26]	Caracas Valley, Venezuela	142–465^c^
Orlova et al. [24]	Moscow, Russia	500–2000^c^
Haugland et al. [14]	Bergen, Norway	91; 39; <5–1779^f^
John et al. [25]	Soshanguve, Pretoria East, South Africa	17.7^g^; 6.9^h^
Berglund et al. [27]	Sweden	410; 480; 610^i^
https://www.epa.gov/superfund/usgs-background-soil-lead-survey-state-data	Washington	26.6; 6.1–513.0^j^
	Oregon	14.4; 2.7–44.7^j^
	Idaho	21.2; 5.5–158^j^

^a^Mean (standard deviation); range.

^b^Mean (standard deviation); median; maximum.

^c^Range.

^d^Playground soil.

^e^Building perimeter soil.

^f^Mean; median; range.

^g^Average. Soshanguve.

^h^Average. Pretoria East.

^i^Individual concentrations reported.

^j^Mean; range.

**Fig. 1:**
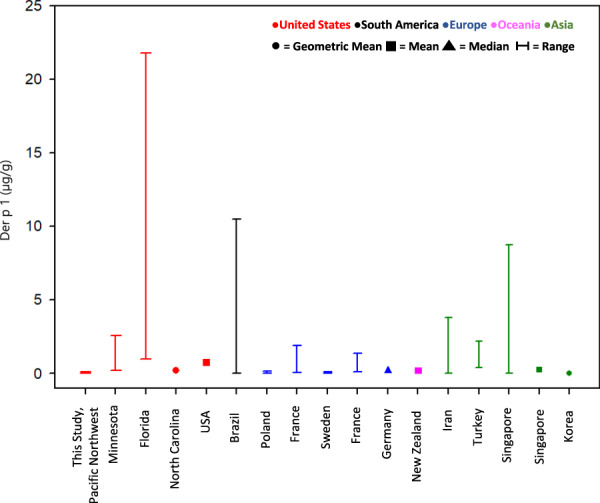
Comparison of Der p 1 concentrations measured in dust collected in childcare centers around the world. Available data are plotted as provided by study authors. See SI for more information.

**Fig. 2:**
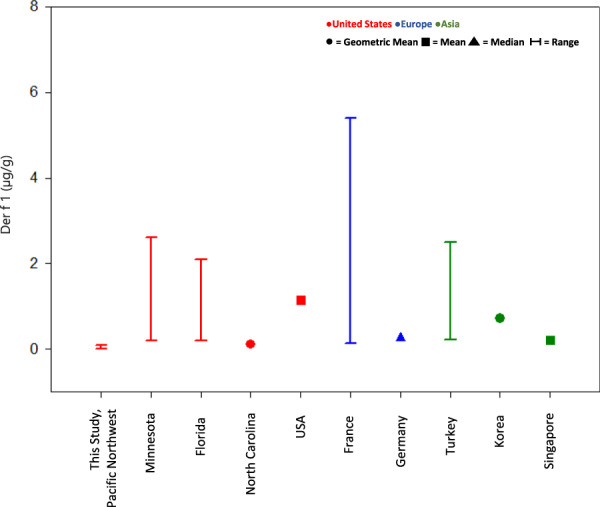
Comparison of Der f 1 concentrations measured in dust samples collected in childcare centers around the world. Available data are plotted as provided by study authors. See SI for more information.

Unlike the pesticide data where there are very few studies available for comparison, there is a plethora of allergen data reported for childcare centers from around the world. Figures 1 and 2 compare Der p 1 and Der f 1 concentrations measured in dust samples collected from childcare centers around the world. Table S9 provides more details including the references, geographic locations, and reported concentrations. The concentration of Der p 1 measured in the childcare centers located in the Pacific Northwest ranged from <0.012–0.12 µg/g (Table S9). In addition to our study, there were four other studies that measured Der p 1 levels in childcare centers in the United States. Our reported study concentrations were less than the other reported measurements. When comparing the Der p 1 levels measured in our study with Der p 1 levels measured in international locations, our concentrations were comparable to the concentrations reported for childcare centers in parts of Europe, Oceania, and Asia (Poland (0.005–0.16 µg/g) [44], Sweden (<0.016–0.106 µg/g) [42], Germany (0.2 µg/g) [46], New Zealand (0.22 µg/g) [49], Singapore (0.3 µg/g) [35], and Korea (0.04 µg/g) [54]). For those locations where Der f 1 was measured, the results show that our reported concentrations (<0.012–0.09 µg/g) are comparable to concentrations reported for childcare centers in North Carolina (0.1 µg/g) [34], Germany (0.26 µg/g) [46], and Singapore (0.2 µg/g) [35] (Fig. 2, Table S9). Geographic location and climatic conditions, including temperature and humidity, may influence dust mite concentrations found in the indoor environment and may explain why it appears that dust mite allergen concentrations are dependent on location [31].

We also measured Bla g 1 (<0.16 Unit/g) and Mus m 1 (<0.001–10 µg/g) in the vacuum dust samples collected in the childcare centers. Very few studies reported concentrations of these allergens in childcare centers in either the United States or globally. The few studies available for comparison are shown in Tables S10 (Bla g 1 comparison) and S11 (Mus m 1 comparison).

The main strength of this study was the collection of a suite of chemical and biological agents from childcare centers located in the Pacific Northwest. To the best of our knowledge, this is the first effort to collect data on several chemical and biological agents from childcare centers located on tribal lands. This study was made possible through established relationships between the Indian Health Service, the Northwest Portland Area Indian Health Board, the 43 federally recognized tribes served by these organizations, and the U.S. EPA. By partnering with the Indian Health Service, the U.S. EPA was able to collect and analyze dust and soil samples for selected pesticides, PCBs, allergens, and Pb, and provide resources for regular use at the childcare centers to reduce potential exposures to these chemical and biological agents including toolkits containing vacuums with HEPA filters. Another advantage of this collaboration was visiting the childcare centers during routine site visits to the centers. This allowed the U.S. EPA and Indian Health Service to maximize resources to better serve the childcare centers. More information on the federal collaboration is available in the SI.

While this study had several scientific strengths, there are limitations to note. These limitations include a small sample size, analysis of limited chemical and biological analytes, one time sample collection, and limited information on pesticide usage patterns. Thirty-one childcare centers participated in this study. The centers that participated represent a convenience sample and what we learned is not generalizable to other childcare centers, even centers with similar demographics. Additionally, the chemical and biological agents included in this study were chosen such that the loadings and concentrations could be compared to the chemical and biological agents measured in the U.S. nationally representative study. Future efforts should include phthalates, flame retardants, and per- and polyfluoroalkyl substances to better understand contemporary compounds to which people are routinely exposed in their everyday environments. Dust and soil samples were collected during one sampling event in the late spring/early summer. We do not know if the analyte concentrations would be different if sampling occurred in other seasons of the year. Another limitation was the low completion rate of the questionnaire administered to the center director (or designee) on selected questions. Information on pesticide use was not adequately collected using this questionnaire because the centers did not document when, where, or who applied the pesticide(s).

Our study reported on selected pesticides, PCBs, allergens, and Pb to better understand the environmental health of childcare centers where students, faculty, and staff may spend significant amounts of time when away from home. By understanding the presence and loadings or concentrations of various chemical and biological agents found in the indoor environment, we can better understand how to prevent/reduce exposures to these agents. Mitigation strategies depend on which chemical and biological agents are present in the indoor and outdoor environments and the potential for exposure to those agents. For example, daily cleaning strategies reduce the amount of dust to which children and adults can be exposed which reduces their potential exposures to chemical and biological agents found in dust.

## Disclaimer

This manuscript has been subjected to Agency administrative review and approved for publication. Mention of trade names and commercial products does not constitute endorsement or recommendation for use. The views expressed in this manuscript are those of the authors and do not necessarily represent the views or policies of the U.S. Environmental Protection Agency or the Indian Health Service. It has been reviewed and approved by the Indian Health Service Institutional Review Board.

### Supplementary Information

Supplementary Information

SUPPLEMENTARY INFORMATION

Supplementary Information

## Data Availability

Analysis information, quality control data, and data comparisons are available in the supplementary information. Additional data are available from the corresponding author on reasonable request.
