# Supplementary material for: Pesticide, allergen, PCB, and lead measurements in childcare centers located on tribal lands in the Pacific Northwest, United States
Source: J Expo Sci Environ Epidemiol. 2023 Sep 11;34(2):217–28. doi: 10.1038/s41370-023-00602-5 (PMC11142913; doi:10.1038/s41370-023-00602-5)
Supplement: Supplementary file 2 — SUPPLEMENTARY INFORMATION [file 41370_2023_602_MOESM2_ESM.docx]

**SUPPLEMENTARY INFORMATION**

PESTICIDE, ALLERGEN, PCB, AND LEAD MEASUREMENTS IN CHILDCARE CENTERS LOCATED ON TRIBAL LANDS IN THE PACIFIC NORTHWEST, UNITED STATES

Nicolle S. Tulve^1,*^, Carry W. Croghan^1^, Bethany L. Plewe^2^, Holly Thompson Duffy^3^, Katie Adams^2^, Theresa McBride^2^, Christopher Pace^2^, Doug Wood^2^, Christopher Fish^4^

^1^United States Environmental Protection Agency, Office of Research and Development, Research Triangle Park, NC

^2^United States Environmental Protection Agency, Region 10, Seattle, WA

^3^Northwest Portland Area Indian Health Board, Portland, OR

^4^Indian Health Service, Portland, OR

*corresponding author: Nicolle S. Tulve, Ph.D., U.S. EPA, Office of Research and Development, Center for Public Health and Environmental Assessment, 109 TW Alexander Dr., MD-E205-04, Research Triangle Park, NC 27711; phone: (919)541-1077; fax: (919)541-4324; email: [tulve.nicolle@epa.gov](mailto:tulve.nicolle@epa.gov)

**METHODS**

**Room Selection**

Classrooms and multipurpose rooms in each participating childcare center were selected for sampling by first conducting an inventory of all classrooms and multipurpose rooms where children <6 years of age regularly spent time. Depending on the number of each type of room in the center, up to two classrooms and two multipurpose rooms were randomly selected. The field technician recorded information about each classroom and multipurpose room on individual inventory sheets and assigned a number to each room found in the center. If the center had more than three classrooms or multipurpose rooms then a die was rolled to determine which classroom or multipurpose room would be sampled. Participating centers had fewer than seven classrooms or multipurpose rooms that qualified for sample collection.

**Room Observations**

Room observations included number of stories and where the room was located, flooring type, temperature, humidity, wall height, room dimensions, number of windows and doorways, window coverings, materials of toys, and evidence of food debris.

**Surface Wipe Sample Collection**

Surface wipe samples were collected following ASTM E1728-16: Standard Practice for Collection of Settled Dust Samples Using Wipe Sampling Methods for Subsequent Lead Determination. This protocol includes first wiping in a horizontal direction within the bounds of the template followed by wiping in a vertical direction within the template followed by wiping along the perimeter of the template. At each change in direction, the dust-collected side of the wipe was folded inward such that a clean side of the wipe was used for each subsequent direction.

**Incremental Sampling Methodology (ISM) (Composite) Soil Sample Collection**

ISM is a structured composite sampling and processing protocol used to provide a reasonable estimate of a chemical’s mean concentration for the volume of soil being sampled. The stainless steel coring tool collected soil plugs of 2.5 cm X 2.5 cm. The coring tool was cleaned with isopropanol and disposable cloths between composite sampling locations.

**Pesticide Analysis by GC/MS:**

| ***Instrument Conditions:*** | ***Injector:*** | ***Column:*** |
| --- | --- | --- |
| Mode: electron impact (EI) | Mode: splitless | Agilent J&W 30 m x 0.25 mm id x 0.25 µm film thickness DB5-5MS UI |
| Source temperature: 300 °C | Volume: 1 µL | Carrier gas: Helium |
| Quad 1 temperature: 150 °C | Pressure: 7.1 psi | Flow rate: 1.2 mL/min |
| Quad 2 temperature: 150 °C | Temperature: 250 °C | Average velocity: 40 cm/sec |
| Collision cell helium quench gas: 2.25 mL/min |  | Oven: 50 °C for 1 min, 20 °C to 325 °C, hold 3 min |
| Collision cell nitrogen collision gas: 1.50 mL/min |  | Run time: 17.75 min |
| Transfer line: 280 °C |  |  |

**PCB Analysis by GC/ECD:**

| ***Instrument Conditions:*** | ***Injector:*** | ***Column 1:*** | ***Column 2:*** |
| --- | --- | --- | --- |
| Mode: Dual electron capture detectors (ECD) | Mode: Pulsed splitless | Restek 15 m x 0.32 mm id x 1.0 µm film thickness Rxi-5ms | Restek 15 m x 0.32 mm id x 0.5 µm film thickness Stx-CLPesticides |
| Detector temperatures: 340 °C | Volume: 1 µL | Carrier gas: Helium | Carrier gas: Helium |
| Makeup gas: Nitrogen | Temperature: 280 °C | Mode: Constant pressure | Mode: Constant pressure |
| Makeup flow: 50 mL/min | Pressure: 7.1 psi | Flow rate: 2.1 mL/min | Flow rate: 2.2 mL/min |
|  | Pulse pressure: 20 psi | Average velocity: 45 cm/sec | Average velocity: 45 cm/sec |
|  | Pulse time: 1 min | Oven: 100 °C for 0.5 min, 20 °C to 200 °C for 0 min, 10 °C to 320 °C, hold 3 min | Oven: 100 °C for 0.5 min, 20 °C to 200 °C for 0 min, 10 °C to 320 °C, hold 3 min |
|  | Purge time: 0.8 min | Run time: 20.5 min | Run time: 20.5 min |

**Lead Analysis by ICP-MS:**

| ***Instrument Conditions:*** |  |
| --- | --- |
| Nebulizer: Micromist | RF Matching: 1.8 V |
| Peltier-cooled Scott-type spray chamber at 2°C | Nebulizer gas flow: 0.65 L/min |
| RF Power 1550 W | Make-up/Dilution gas (HMI): 0.38 L/min |
| Plasma gas flow: 15 L/min | Sample depth: 8 mm |

Lead values were reported as the sum of Pb206+Pb207+Pb208. Bi 209 used as internal standard. No collision or reaction gases were used for the analysis of lead.

**Examples: Reporting Limit Calculations**

Wipe Samples for Pesticides

(1 ng/mL) x (2 mL) = 2 ng per wipe sample

where 1 ng/mL is the low point on the calibration curve and 2 mL is the final volume.

Wipe Samples for PCBs

(25 ng/mL) x (2 mL) = 50 ng per wipe sample

where 25 ng/mL is the low point on the calibration curve and 2 mL is the final volume.

Soil Samples for Pesticides

(1 ng/mL) x (4 mL)/(10 g) x (% S) = 0.40 µg/kg

where 1 ng/mL is the low point on the calibration curve, 4 mL is the final volume, 10 g is the sample weight, and % S is the percent solids in decimal format.

Soil Samples for PCBs

(25 ng/mL) x (4 mL)/(10 g) x (% S) = 10 µg/kg

where 25 ng/mL is the low point on the calibration curve, 4 mL is the final volume, 10 g is the sample weight, and % S is the percent solids in decimal format.

Wipe Samples for Lead

(0.5 µg/L) x (0.05 L) x 10 = 0.25 µg per wipe sample

where 0.5 µg/L is the demonstrated minimum level of quantitation of lead in a wipe sample, 0.05 L is the final volume, and 10 is the analysis dilution.

Soil Samples for Lead

(0.25 µg/L) x (0.05 L)/(0.5 g) x 10 = 0.25 mg/kg

where 0.25 µg/L is the demonstrated minimum level of quantitation of lead in a soil sample, 0.05 L is the final volume, 0.5 g is the sample weight digested, and 10 is the analysis dilution.

**Spike Concentrations – Pesticides and PCBs**

All wipe samples were spiked with 200 µL of a 1 ng/µL pesticide surrogate solution resulting in 100 ng/mL on-column or 200 ng wipe sample amount with a 2 mL final volume.

LCS/LCSD wipe samples were spiked with 200 µL of a 1 ng/µL pesticide analyte spike solution resulting in 100 ng/mL on-column or 200 ng wipe sample amount with a 2 mL final volume.

All wipe samples were spiked with 40 µL of a 5 ng/µL PCB surrogate solution resulting in 100 ng/mL on-column or 200 ng wipe sample amount with a 2 mL final volume.

LCS/LCSD wipe samples were spiked with 200 µL of a 4 ng/µL PCB analyte spike solution resulting in 400 ng/mL on-column or 800 ng wipe sample amount with a 2 mL final volume.

All soil samples were spiked with 400 µL of a 1 ng/µL pesticide surrogate solution resulting in 100 ng/mL on-column or 40 µg/kg in a 10 g sample with a 4 mL final volume.

LCS/LCSD and MS/MSD soil samples were spiked with 400 µL of a 1 ng/µL pesticide analyte spike solution resulting in 100 ng/mL on-column or 40 µg/kg in a 10 g sample with a 4 mL final volume.

All soil samples were spiked with 80 µL of a 5 ng/µL PCB surrogate solution resulting in 100 ng/mL on-column or 40 µg/kg in a 10 g sample with a 4 mL final volume.

LCS/LCSD and MS/MSD soil samples were spiked with 400 µL of a 4 ng/µL PCB analyte spike solution resulting in 400 ng/mL on-column or 160 µg/kg in a 10 g sample with a 4 mL final volume.

**Spike Concentrations – Lead**

All lead spike samples contained 25 µg of lead delivered via a liquid standard spike which produced a spike concentration of 50 µg/L on-instrument.

LCS and MS/MSD samples for wipe and soil samples were spiked with 2.5 mL of 10,000 µg/L (10 mg/L) lead-containing solution resulting in 50 µg/L lead on-instrument when analyzed at a 10X dilution.

**Regional Screening Levels**

The U.S. EPA has established levels of pesticide concentrations requiring immediate remedial action called Regional Screening Levels (RSLs). More information about RSLs can be found at <https://www.epa.gov/risk/regional-screening-levels-rsls-generic-tables>.

**Signal Word Descriptors**

Signal words describe the acute (short-term) toxicity of a formulated pesticide product (<http://npic.orst.edu/factsheets/signalwords.html>). Signal words may change between products with the same active ingredient due to concentration, formulation, and registered uses. One pesticide, azinphos methyl, was labeled with the signal word “Danger” and is no longer registered for use with the U.S. EPA. Additionally, two other “Danger”-labeled pesticides were detected in this study: chlorothalonil and *lambda*-cyhalothrin.

**Inter-Agency Collaboration with External Partners**

The success of this project on tribal lands relied heavily on leveraging U.S. EPA regional relationships with both the Portland Area Indian Health Service and the Northwest Portland Area Indian Health Board. These project partners enabled the Indian Health Service and the U.S. EPA to combine sampling with education and outreach. Each participating childcare center received an individualized report containing their center-specific measurement results, recommendations on ways to reduce potential exposures to chemical and biological agents found in the centers, and toolkits (e.g., HEPA filtration vacuums) to use in the center. The Portland Area Indian Health Service and Northwest Portland Area Indian Health Board also held follow-up discussions with interested centers to help them establish action plans to implement recommended improvements.
